# A versatile and low-cost open source pipetting robot for automation of toxicological and ecotoxicological bioassays

**DOI:** 10.1371/journal.pone.0179636

**Published:** 2017-06-16

**Authors:** Sebastian Steffens, Leonie Nüßer, Thomas-Benjamin Seiler, Nadine Ruchter, Mark Schumann, Ricarda Döring, Catrina Cofalla, Avi Ostfeld, Elad Salomons, Holger Schüttrumpf, Henner Hollert, Markus Brinkmann

**Affiliations:** 1 Department of Ecosystem Analysis, Institute of Environmental Research, ABBt – Aachen Biology and Biotechnology, RWTH Aachen University, Aachen, Germany; 2 Aquatic Ecology and Centre of Water and Environmental Research (ZWU), University of Duisburg-Essen, Essen, Germany; 3 Institute for Hydraulic Engineering and Water Resources Management, RWTH Aachen University, Aachen, Germany; 4 Environmental, Water and Agricultural Engineering, Faculty of Civil and Environmental Engineering, Technion – Israel Institute of Technology, Haifa, Israel; 5 OptiWater, Haifa, Israel; 6 State Key Laboratory of Pollution Control and Resource Reuse, School of the Environment, Nanjing University, Nanjing, China; 7 College of Resources and Environmental Science, Chongqing University, Chongqing, China; 8 Key Laboratory of Yangtze Water Environment, Ministry of Education, Tongji University, Shanghai, China; Oregon State University, UNITED STATES

## Abstract

In the past decades, bioassays and whole-organism bioassay have become important tools not only in compliance testing of industrial chemicals and plant protection products, but also in the monitoring of environmental quality. With few exceptions, such test systems are discontinuous. They require exposure of the biological test material in small units, such as multiwell plates, during prolonged incubation periods, and do not allow online read-outs. It is mostly due to these shortcomings that applications in continuous monitoring of, e.g., drinking or surface water quality are largely missing. We propose the use of pipetting robots that can be used to automatically exchange samples in multiwell plates with fresh samples in a semi-static manner, as a potential solution to overcome these limitations. In this study, we developed a simple and low-cost, versatile pipetting robot constructed partly using open-source hardware that has a small footprint and can be used for online monitoring of water quality by means of an automated whole-organism bioassay. We tested its precision in automated 2-fold dilution series and used it for exposure of zebrafish embryos (*Danio rerio*)–a common model species in ecotoxicology—to cadmium chloride and permethrin. We found that, compared to conventional static or semi-static exposure scenarios, effects of the two chemicals in zebrafish embryos generally occurred at lower concentrations, and analytically verified that the increased frequency of media exchange resulted in a greater availability of the chemical. In combination with advanced detection systems this custom-made pipetting robot has the potential to become a valuable tool in future monitoring strategies for drinking and surface water.

## Introduction

The aquatic environment in densely inhabited and industrialized areas around the world faces contamination with a plethora of anthropogenic chemicals [1]. Pollutants may not only harm aquatic wildlife; they may also contaminate water bodies intended for drinking water production or water distribution systems, and in this way pose a risk to humans as well [2]. In order to immediately detect contamination of the water distribution system, and ultimately to protect humans from consuming contaminated drinking water, early water quality warning systems have been implemented in many countries around the world [3].

The most widely used type of systems is based on physicochemical measurements to detect sudden changes in water quality [4]. While such systems are relatively inexpensive and do not typically require extensive maintenance, they are also limited in that they are suitable to detect only certain contamination events, and often at fairly high concentrations (e.g., flooding of or seepage into drinking water wells). On the other hand biological early warning systems (BEWSs) may be used, which continuously track the physiological or behavioral reactions of organisms to detect sudden increases of the concentration of contaminants [5, 6]. The development of the first BEWSs dates back to several decades ago. Recent advances in available technology for acquisition and analysis of the reactions of organisms, along with a concurrent reduction in cost of such systems, has leveraged their application in recent years [4]. Furthermore, the assessment of changes in behavior of organisms as a sensitive and rapid indicator of contamination events (e.g., avoidance/attraction, swimming patterns, inter- and intraspecific interactions, ventilation and feeding patterns, etc.) has developed into a highly active field of dedicated scientific research, with a strong potential for application in BEWS [7, 8]. A number of challenges remain, which limit the use of and in particular the regulatory relevance of BEWSs (e.g., the lack of standardization, difficulty in interpretation of readouts, non-monotonous reactions, high variation between individual organisms) [9].

Current testing strategies to identify the environmental risks of chemicals within the given regulatory context rely highly on whole-organism bioassays with, e.g., algae, daphnids and fish [10–13]. These are not only important in compliance testing of industrial chemicals and plant protection products, but also in the monitoring of environmental quality. With few exceptions, such bioassays are discontinuous: they require exposure of the biological test material in small units, such as multiwell plates, during prolonged incubation periods, and do not provide online read-outs. We believe that it is mostly due to these shortcomings that applications in continuous monitoring of, e.g., drinking or surface water quality are largely missing. In recent years, the fish embryo toxicity (FET) test with zebrafish embryos has become an important test system of international relevance [14] due to a number of advantages: zebrafish spawn throughout the year and experiments with embryos/larvae are not legally considered animal experiments up to 120 hours post fertilization by EU legislation [15]. Furthermore, recent developments enabled researchers to conduct experiments for reliable and automated/unattended monitoring of larval behavior, with the potential to detect immediate behavioral changes as an indication for neurotoxic or at least repellent effects. Last, the FET test has been standardized by international organizations and is already used for (offline) monitoring of the quality of effluents of sewage treatment plants in Germany [16–18].

The contrasting demands of (a) performing bioassays in compliance with regulatory testing strategies, and (b) their potential use in online monitoring of water quality call for automation technology to assist with automated handling and analysis of multiwell plates. Such systems are typically highly sophisticated and thus costly. As a consequence, the availability of pipetting robots, liquid handlers, and stacking units in environmental monitoring is generally scarce. As a potential solution, we developed a simple and low-cost, versatile open-source pipetting robot that has a small footprint. Its construction as described in this article can be easily achieved by untrained staff and realized mostly using readily available parts, and partly using open-source hardware. It makes use of commercially available multi-channel pipettes and the very popular open-source rapid prototyping platform Arduino, which—among many other benefits—excels through the ease of use, availability of add-on hardware and publicly available software libraries [19]. To assess the precision of the newly developed robot and its use in water quality monitoring, we performed automated 2-fold serial dilutions with the two dyes neutral red and resorufin. Furthermore, we used it for exposure of embryos of zebrafish (*Danio rerio*) to cadmium chloride and permethrin as model compounds for heavy metals and organic chemicals, respectively.

## Materials and methods

### Chemicals

Resorufin (CAS 635-78-9), neutral red (CAS 553-24-2), permethrin (CAS 52645-53-1), cadmium chloride (CAS 10108-64-2), 2,4-dichloroaniline (DCA; CAS 554-00-7) and dimethyl sulfoxide (DMSO; CAS 67-68-5) were purchased from Sigma Aldrich GmbH (Steinheim, Germany). Stock solutions of permethrin were prepared and diluted in DMSO, to ensure that all exposure concentration contained the same concentration of DMSO (0.1%). Stock solutions of cadmium chloride were prepared directly in test medium.

### Pipetting robot

#### Construction

**Fig 1:**
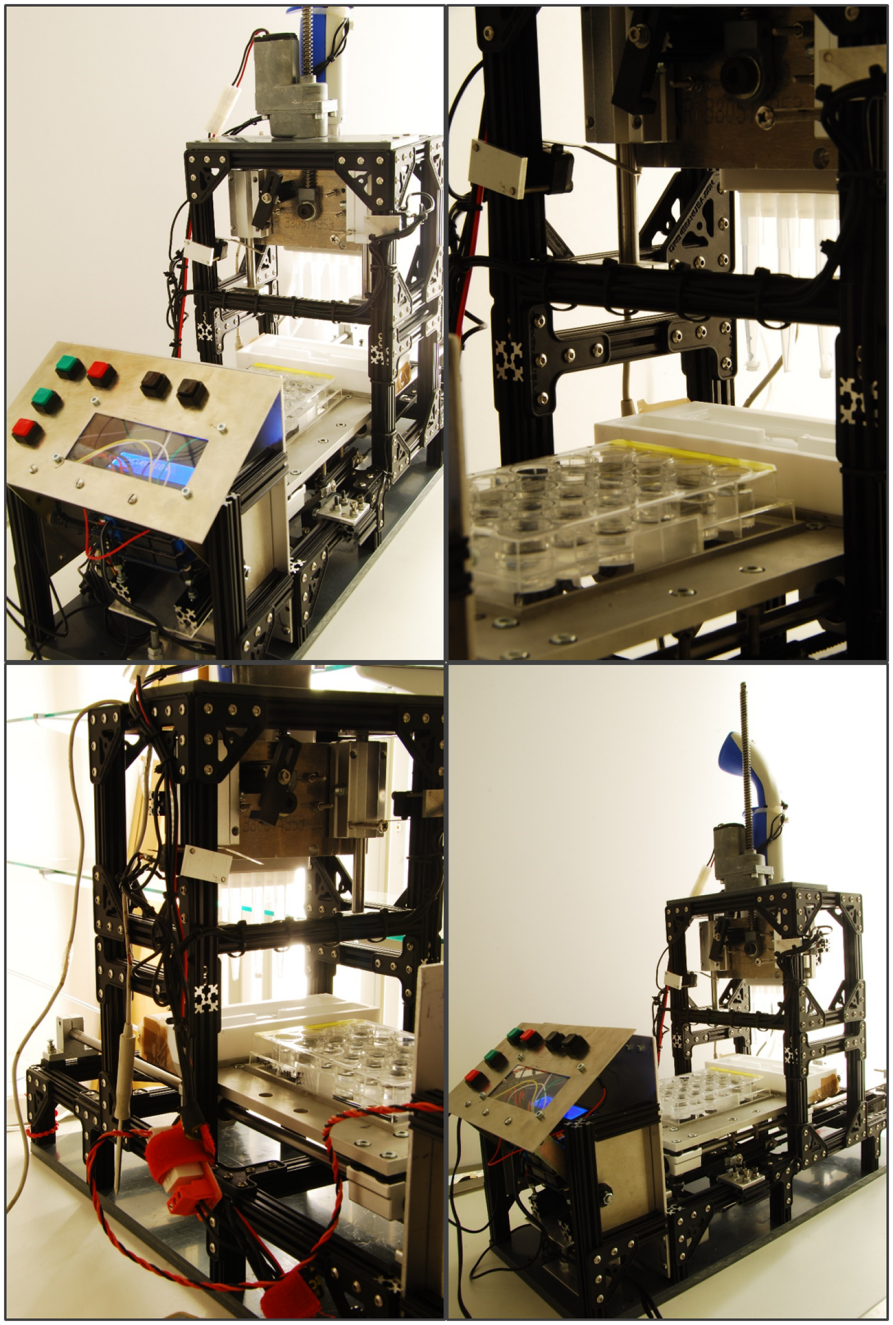
Photographs of the pipetting robot. The depicted pipetting robot was constructed for and used in the present study.

The pipetting-robot used in the present study (Fig 1) was constructed of components readily available by a number of suppliers, with a minimum number of customized parts. A complete list of components and technical drawings of the customized parts is available in the Supplementary Information (Table A in S1 File). The base frame was constructed using aluminum extrusion profiles purchased from OpenBeam USA (www.openbeamusa.com) and mounted on a polyvinylchloride (PVC) baseplate (Fig A in S1 File. An electronic eight-channel pipette (BioPette E; Labnet International Inc., Edison, USA), which was attached to a customized steel plate (Fig B in S1 File) can be moved vertically on linear ball bearings (Adafruit Industries, New York, USA) along two stainless steel shafts using a spindle drive. A customized steel moving platform (Fig C in S1 File) was equipped with a recess to accommodate a standard multi-well plate, and additional recesses for sample intake and outflow (Fig D in S1 File). This part was manufactured from polytetrafluoroethylene (PTFE) to prevent any interactions between the vessel material and the investigated substances. Driven by a stepper motor (Adafruit, NEMA, 12V, 350 mA) and a timing belt, the moving platform was designed to horizontally move on linear bearings along two stainless steel shafts. As such, the electronic multi-channel pipette is able to reach all wells of the multi-well plate, as well as the sample intake and outflow. Micro-switches at the end of both axes (horizontal and vertical) are used to restrict the movement of the pipette and to reset the position of the moving platform along their axes, respectively.

The control electronics as well as the programming are based on the open-source prototyping platform Arduino (www.arduino.cc; Creative Commons Attribution Share-Alike license). The central control element is the Arduino UNO rev. 2 microcontroller board. Each motor of the robot is controlled by a motor shield (Adafruit, motor/stepper/servo shield for Arduino v2 kit). Another shield (Adafruit, LCD shield kit with 16x2 characters) equipped with a liquid crystal display (LCD) and a simple keypad allows for the selection of different programs and interaction with the control elements. To control the eight-channel pipette *via* the Arduino board, the pipette’s electronics (push button for automatic aspiration and discharge) were hard-wired to a digital pin of the control element and the battery replaced with a plug-in power supply. The eight-channel pipette had been equipped with a sleep mode that gets by default activated after 10 min to save on battery. To prevent the sleep mode from interfering with prolonged experiments, the pipette is restarted after each incubation cycle using an Arduino-controlled relay module (TinkerKit; www.tinkerkit.com). The standard pipetting volume was set to 1 mL per channel.

#### Testing the performance of the pipetting robot

Two different programs were implemented for the pipetting robot: (a) a program for two-fold serial dilutions (mainly with the intention to test the accuracy of the unit), and (b) a program for automated exchange of exposure media, to perform semi-static bioassays (S2 file: Arduino sketches).

The pipetting robot’s ability to perform serial dilution-series was tested within a 24-well microplate using only four of the eight available channels. Serial dilutions of resorufin (starting at 2.4 μg L^-1^) and neutral red (starting at 200μg L^-1^) were performed as pre-tests. Highest used concentrations were 200 μg L^-1^ for neutral red and 2.4 μg L^-1^ for resorufin, respectively. To this end, 2mL of a solution of either 2.4 μg L^-1^ resorufin or 200 μg L^-1^ neutral red in water were added to all wells of the first column of the plate. The remaining wells received 1 mL distilled water each. The robot aspired 1 mL from the first column, discharged it in the next column and thoroughly mixed the solution by repeated aspiration and discharge (four times) before transferring it to the next column. Triplicate dilution series with four technical replicates each were prepared for both chemicals tested. Fluorescence of resorufin (excitation: 560 nm, emission: 590 nm) and absorption of neutral red at 540 nm were measured using a spectrofluorometer (Infinite M200, Tecan, Männedorf, Switzerland). Resulting data were analyzed by linear regression.

**Fig 2:**
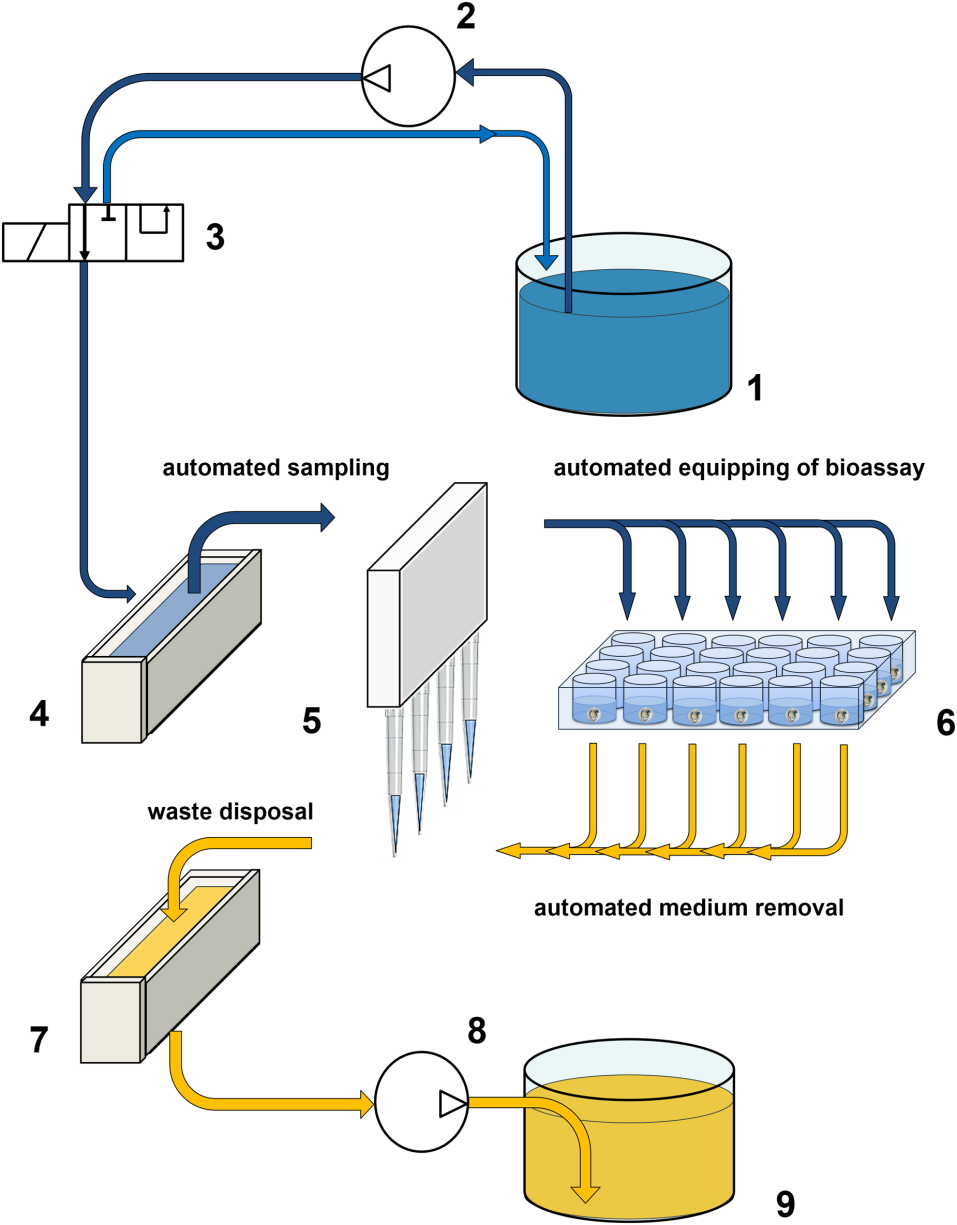
Process for automated exchange of exposure media. Flow diagram of the process utilized to automatically exchange exposure media in the fish embryo toxicity (FET) test.

The flow diagram of a program for automated exchange of media in bioassays is shown in Fig 2. The eight-channel pipette (5) was used to remove 50% (1 mL) of exposure medium per well (6) and dispose it in the outflow (7). The discarded medium was pumped into the waste container (9) by use of a peristaltic pump (8; Masterflex 7015, Merck Millipore, Darmstadt, Germany; with Tygon^®^ tubing, Saint-Gobain Performance Plastics, Paris, France). Fresh medium was pumped from the storage tank (1) to the PTFE sample inflow (4) by use of a peristaltic pump (2; 205s, Watson Marlow GmbH, Rommerskirchen, Germany). In case of an overflow event in the sample inflow, the medium automatically flows into the outflow. The pipette was programmed to transfer 1mL of fresh medium from the sample inflow into each well of the microplate. After refilling the plate was incubated for a user-specified time before the next exchange cycle started. During this incubation time a three-way valve (3) was operated to short-circuit the inflow from the storage tank (1). This approach prevented use of unnecessary large amounts of media. The program was intended for application in 24-well and 6-well microplates. All tubing delivering exposure solutions to the exposure unit (except for the Tygon^®^ tubing used in the peristaltic pump) consisted of fluorinated ethylene propylene (FEP) or PTFE.

### Fish embryo toxicity (FET) test

#### Fish culture

A breeding stock of zebrafish was held in glass aquaria according to culture conditions previously defined [20–23]. Fish were cultivated in standardized water at pH 7.0 ± 0.2, and temperature was maintained at 26°C ± 1°C. Water was constantly filtered in a recirculating system with continuous water exchange at a rate of approx. 50% per week. Adult fish were kept in groups of approx. 20 individuals with a sex-ratio of approx. 3:2 (male: female), under a constant artificial dark/ light cycle of 10/14 h. Zebrafish were daily fed *ad libitum* with dry flake food (Tetra Min^®^; Tetra GmbH, Melle, Germany) and with living nauplius larvae of *Artemia sp*. (Silver Star Artemia; Inter Ryba GmbH; Zeven, Germany) in the afternoon before spawning. Glass spawning trays covered by a stainless steel with attached artificial plants were placed into the aquaria in the afternoon before spawning. Mating, spawning and fertilization took place within 30 min after the onset of illumination in the morning. Spawning trays were removed after two hours and eggs were microscopically selected.

All experiments conducted in the present study were in accordance with the Animal Welfare Act and performed with permission of the federal (Landesamt für Natur, Umwelt und Verbraucherschutz NRW, Germany) and local (Amt für Verbraucherschutz, Tierschutz und Veterinärwesen, Städteregion Aachen, Würselen, Germany) authorities. Since none of the larvae used in the present study had an age of equal to or greater than 120 hpf (hours post fertilization), the experiments conducted within the present study are not legally considered animal experiments within the European Union [24]. For the ease of reading, however, we chose to use 120 h to describe the last time point of exposure. No adult zebrafish and/or zebrafish older than or equal to 120 hpf were used.

#### Selection of eggs for use in the FET test

Selection of eggs and the FET test were conducted according to DIN 38415 [16]. Fertilized zebrafish eggs were inspected visually using a binocular microscope (SMZ 1500, Nikon GmbH, Düsseldorf, Germany). Only normally developed fish eggs were chosen for further testing. The selected eggs had to be developed at least to the 8-cell stage and not exceeding the 64-cell stage. Afterwards, the eggs were transferred into formulated water that was prepared according to the guideline. To ensure sufficient oxygen saturation, the formulated water was aerated for 24 h prior to usage.

#### Pre-tests

Before experiments with the investigated chemicals were conducted, it was verified that the pipetting robot had no negative effects on fish embryos. Development and mortality of a negative control group incubated using the robot was compared to an external, static negative control. The robot was used to automatically exchange 50% of the medium within the plate in 1h intervals. All tests were performed in 24-well microplates, placing five eggs in 2 mL of medium per well. Eggs were exposed for up to 120 hpf and microscopically inspected every 24 h using an inverted microscope at 40-fold magnification (Eclipse TS100, Nikon GmbH). Lethal and sub-lethal endpoints, as well as the hatching rate were determined in three independent replicates. Survival rate and hatching rate of the two groups were statistically evaluated using Student’s *t*-test (*p = 0*.*05*).

#### Control groups

To ensure validity of the conducted FET tests, different control groups were investigated during each experiment. For the extended exposure time of up to 120 hpf, the following validity criteria were adapted from OECD Guideline 236 [17]: for each test, the survival rate of unexposed negative control animals had to be ≥ 90% at the end of the test, and the hatching-rate had to exceed 80% after 96 hours. If the selected endpoint for the FET test was mortality, a positive control containing 3.7 mg L^-1^ DCA was investigated. Mortality in the positive control had to exceed 10% after 48 h and 30% after 96 h exposure. Permethrin was dissolved in dimethyl sulfoxide (DMSO; Sigma-Aldrich); thus, a solvent control was investigated containing the same concentration of DMSO as used in the test (0.1%).

#### Exposure of fish embryos

The FET test with each of the model substances was performed under three different exposure conditions. The substances where tested in a static FET test according to the DIN/ OECD guidelines, in a semi-static test in which exposure media were exchanged every 24 h, and using the pipetting robot in which 50% of the exposure media were exchanged in 1 h intervals using only four of the eight available channels of the pipette. Three independent replicates were investigated using the static and semi-static exposure designs, while each concentration was tested once using the robot. Since it was not possible to change the volume setting of the integrated eight-channel pipette during operation, only one concentration of each substance was tested per well plate in a separate experiment, each of which was accompanied by the required control groups. Static and semi-static exposures were incubated at 26°C ± 1°C. FET tests utilizing the pipetting robot were performed in a climatic chamber set to 26.0°C (measured temperature range: 24.9°C to 27.6°C). To prevent the eggs from getting aspirated by the eight-channel pipette of the pipetting robot, the opening of wide tips (Labomedic GmbH, Bonn, Germany) were covered using 200μm mesh PTFE gauze. The gauze was fixed to the pipette tip using a shortened piece of another pipette tip. While the FET test was conducted according to DIN 38145, the protocol was slightly modified to account for the investigated substances through sealing multiwell plates with gas-permeable adhesive foil (VWR International GmbH, Darmstadt, Germany) during incubation to prevent evaporation of media. Plates exposed within the robot were not sealed.

For tests with permethrin, exposure of all eggs was started simultaneously through pre-incubation in 70 mL glass crystalizing dishes (VWR International GmbH, Darmstadt, Germany) and afterwards the eggs were transferred to 24-well plates which were pre-conditioned with the test solutions for 24 h prior to the test to saturate potential binding sites with the chemical. One embryo was incubated in 2 mL of each solution per well. For the static and semi-static tests, 10 embryos were used per concentration (12 concentrations ranging from 0.025 to 0.9 mg L^-1^), 40 embryos for the negative control and 10 for the solvent control. For exposure using the pipetting robot, 24 eggs were used for each concentration and each control group. In addition to the endpoints defined by the FET test guidelines, two additional sub-lethal endpoints were recorded up to 120 hpf: (a) the occurrence of uncontrolled muscle convulsions and (b) the inability of the larvae to maintain a normal upright body posture (hereafter referred to as “impaired swimming”). To record both endpoints, each larva was observed for 20 s and only surviving and hatched lavae were included in the analysis. Since no reference substance was available for these endpoints, no positive controls were investigated for permethrin.

Tests with cadmium chloride were performed in 6-well plates (VWR International GmbH, Darmstadt, Germany), where 5 embryos were exposed in 10 mL per well. Eggs contained within 1 mL of artificial water were directly transferred into 9 mL of previously prepared solution per well. Pre-exposure in glass crystallization dishes was thus unnecessary, thereby reducing the risk of binding of cadmium to glass surfaces. Ten eggs were exposed to each cadmium chloride test concentration (six concentrations ranging from 2.50 to 80.0 mg L^-1^ cadmium chloride, or 1.56 to 49.1 mg L^-1^ cadmium equivalents). Forty eggs were used for the negative control and 20 for the positive control of each experiment. For testing the robot, 36 eggs per concentration were exposed in 6-well plates. The control groups also consisted of 36 eggs each. The investigated endpoint was the mortality after 48 h exposure.

Embryos were microscopically inspected every 24 h for the previously listed lethal and sub-lethal endpoints.

### Chemical analysis of exposure concentrations

Chemical analysis of actual exposure concentrations of permethrin and cadmium (Cd) was performed by sampling test solutions from the multiwell plates at various time points. A single concentration close to the semi-static EC_50_ of the two chemicals was chosen for exposure. Analytically determined concentrations of initial concentrations of permethrin and cadmium in exposure working solutions were 25 μg L^-1^ (manual changes) and 29 μg L^-1^ (robot) for permethrin, and 31.8 mg L^-1^ (2-fold concentrated, manual changes) and 19.1 mg L^-1^ (robot) for Cd, respectively. Samples were taken once daily for static exposure and exposure in the robot, while four samples per day were taken for semi-static exposure. The solutions from four individual wells were combined into one sample. Three technical replicates were sampled per treatment and time point.

For permethrin samples, *trans*-Permethrin-(*phenoxy*-d_5_) (Sigma Aldrich) was added as an internal standard prior to liquid-liquid extraction with 1 ml toluene. Extracts were then analyzed using an Agilent Technologies GC system (7890 A GC System and 5975 C inert XL MSD with Triple-Axis-Detector, Agilent Technologies Deutschland GmbH, Böblingen, Germany).

For Cd analysis, samples were acidified with nitric acid prior to storage. Concentrations of Cd were analyzed by electrothermal atomic absorption spectrometry (ET-AAS) using a Perkin-Elmer Zeeman effect background correction system (Perkin-Elmer, Massachusetts, USA). Samples were diluted 40,000-fold with ultrapure water prior to analysis. An autosampler AS70 was used to inject 20 μL of the diluted samples into the furnace unit. Each subsample was analyzed in triplicate.

Five calibration standard solutions were prepared (0, 0.5, 1; 2 and 5 μg L^-1^) diluting a Cd AAS-Standard solution (Bernd Kraft GmbH, Germany) with ultrapure water to achieve a linear regression line with a correlation coefficient > 0.99. Concentrations of the samples were interpolated from these regression lines. For quality assurance, twelve procedural blanks were prepared from the standard water used in the experiment and handled like the samples (40,000-fold dilution). The limit of detection was calculated as the threefold standard deviation of the blank measurements. This resulted in a limit of detection (LOD) of 0.9 mg/L. Furthermore, ten procedural blanks were spiked with a Cd standard solution of 0.5 μg L^-1^ and ten further procedural blanks were spiked with a Cd standard solution of 5 μg L^-1^. Analysis resulted recovery rates of 0.4 ± 0.3 μg L^-1^ and 4.0 ± 0.37 μg L^-1^, respectively.

### Statistical analysis

All spreadsheet calculations of lethal and sublethal endpoints were performed using Microsoft Excel^®^ 2013. Data was evaluated and plotted using GraphPad Prism 5 software (GraphPad Software Inc., La Jolla, USA). Concentration-response curves were fitted *via* four parameter logistic regression with top and bottom values set to 100% and 0%, respectively. Resulting LC_50_ and EC_50_ values were interpolated from the regression curves. At the present stage, the pipetting robot could only test one concentration of each substance at a time. To allow for investigation of concentration-response relationships, the resulting data were merged into one data set. A separate control group was investigated in each experiment. The arithmetic mean of all the control groups was calculated from all experiments with one substance. To allow for straight-forward comparison between concentration-response curves of conventional FET tests and experiments using the pipetting robot, three data points each of concentration-response curves generated using the pipetting robot, and the manually conducted semi-static experiments, respectively, were compared using the χ^2^-test (*p = 0*.*05*, two-tailed).

## Results and discussion

### Dilution series

**Fig 3:**
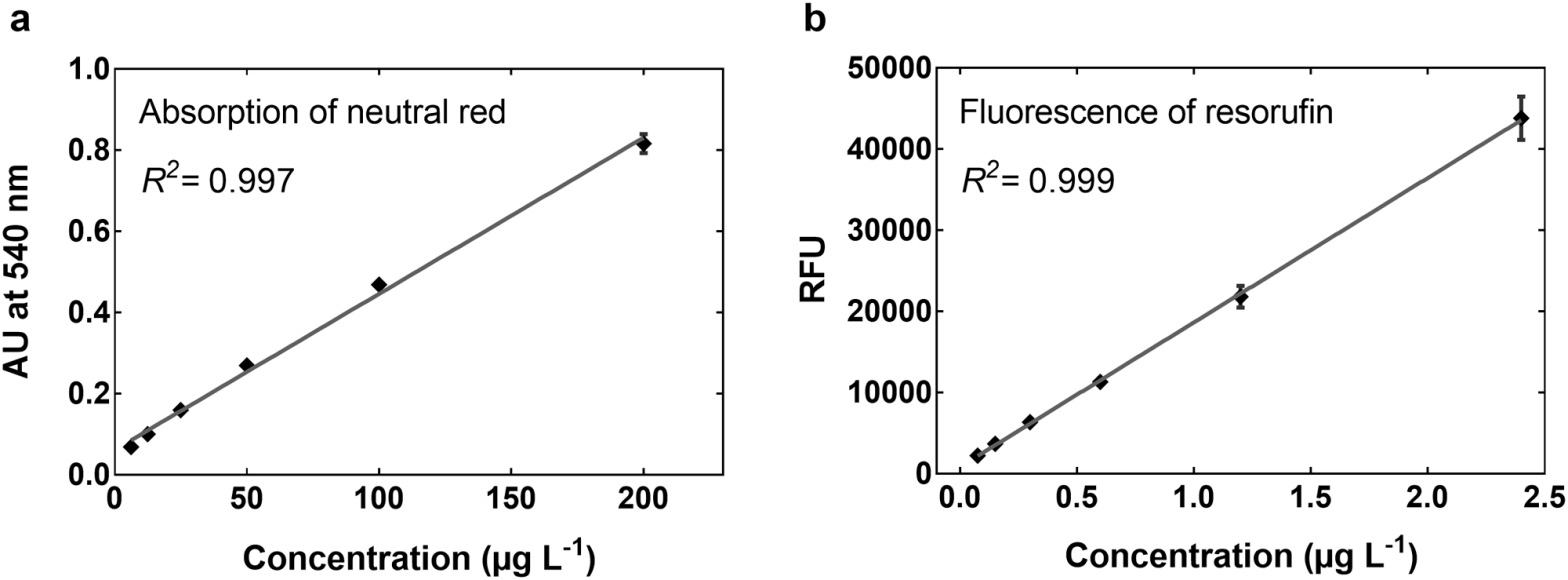
Linear regression analysis of the serial dilution series pipetted by the robot. Absorption of neutral red (left) and fluorescence of resorufin (right) were measured by use of a spectrofluorometer. Data points indicate mean ± standard deviation of *n* = 3 independent dilution series.

Accuracy and reproducibility of the pipetting steps performed by the pipetting robot are of vital importance for valid and reliable outcomes of any downstream bioassays. Due to the fact that the current design concept did not involve any modification of the pipette’s hardware, in the present setup, accuracy and reproducibility do not deviate from the manufacturer’s specifications. To test if the sequence of pipetting steps performed by the robot yields precise outcomes, two commonly used dyes, neutral red and resorufin, were subjected to 2-fold serial dilution (*n* = 3). The absorption of neutral red and the fluorescence of resorufin were measured by using a spectrofluorometer and analyzed by means of linear regression (Fig 3). For both substances, the resulting coefficients of determination (*R*^*2*^) indicated a strong linear correlation and a high precision of the pipetting steps.

These data show that the sequence of steps involved in manipulating the volumes of liquids within the multiwell plates (e.g., aspiration and dispensing steps, mixing, movements along the vertical and horizontal axes) yielded precise results. Based on these data, it can be assumed that simpler pipetting tasks, such as water exchanges in multiwell plates, will be performed with the same success by the pipetting robot.

### Influence of the pipetting robot on survival and hatching rate

**Fig 4:**
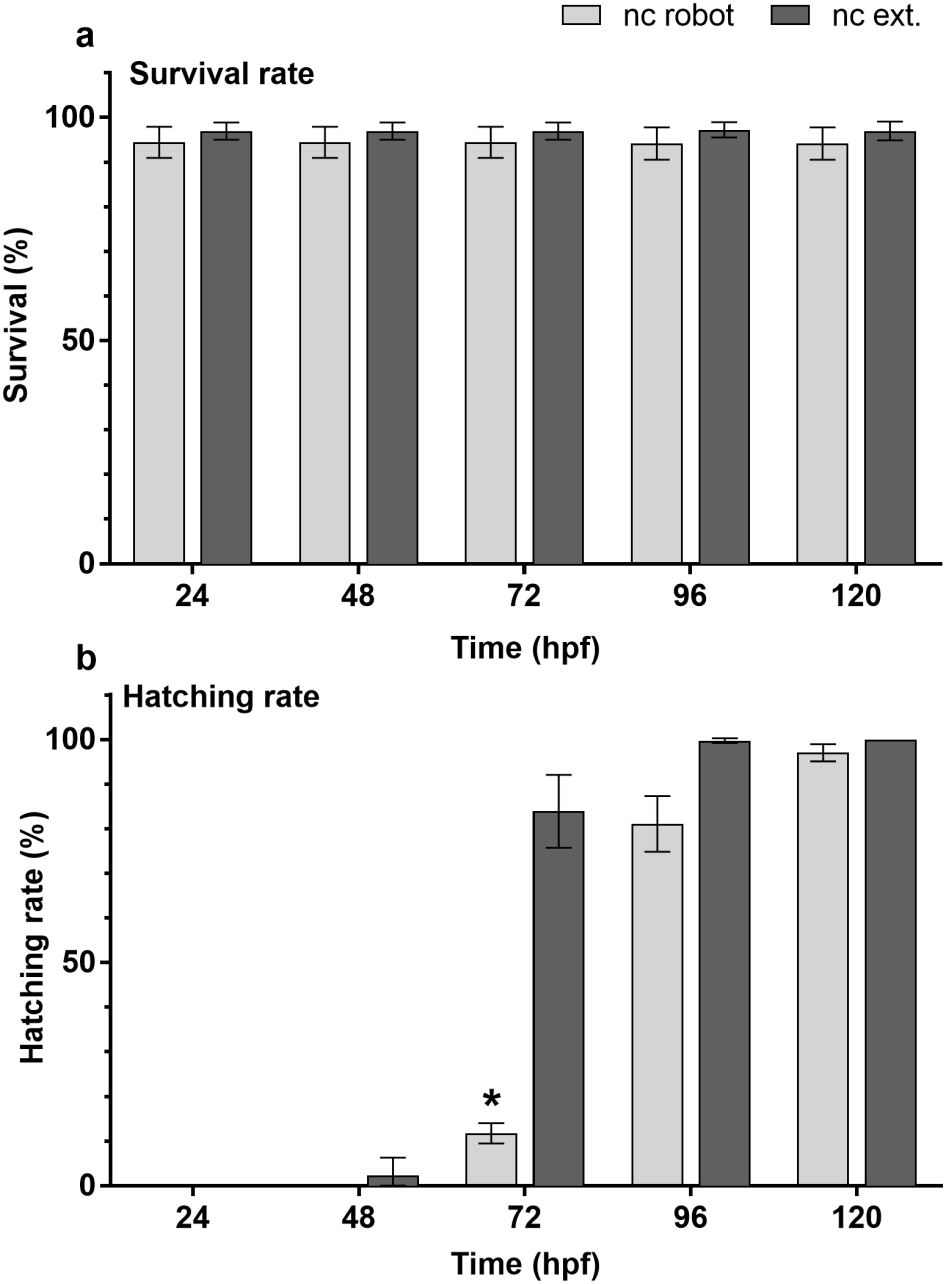
Comparison of the survival and hatching rates. Survival (top) and hatching rates (bottom) were compared between control groups incubated in the robot (nc robot) and external control group (nc ext.) after 24, 48, 72, 96 h exposure and up to 120 hpf. Bars represent mean ± standard deviation from *n* = 3 independent replicates. Asterisks indicate statistically significant differences for each time point (Student’s *t*-test, *p* ≤ 0.05).

In order to determine if handling by the robot had significant effects on survival and hatching rate of the incubated embryos, a pre-test was conducted in a climatic chamber set to 26°C (measured air temperature range: 24.9°C to 27.6°C). Survival rates of embryos when exchange of exposure medium was performed by the robot as well as that of embryos in external control groups were greater than 90% at all times (Fig 4). No statistically significant differences were observed between survival of both groups (Student’s t-test, *p* > 0.05). Since survival rates exceeded 90% in all replicates and for both investigated groups, these tests were valid according to OECD guideline 236 [17].

A slightly delayed hatching of zebrafish embryos was observed in control groups incubated using the pipetting robot (Fig 4). While the external control group started hatching already after 48 h, the group handled by the robot began to hatch after 72 h, leading to a significant difference after 72 h (Student’s *t*-test, *p* ≤ 0.05). While the hatching rate of the embryos handled by the robot was approx. 12%, already 84% of the embryos within the external control group were hatched. Hatching rates approximately equaled after up to 120 hpf. Although the hatching rate after 96 h was slightly lower (not significant) for the embryos handled by the robot compared to that of the external control group, both tests would have been considered valid according to OECD guideline 236, which demands that ≥ 80% of the embryos have hatched after 96 h. A slight temperature difference (approx. 1.0°C) between the moving platform of the robot and the surrounding incubation chamber was recorded, which was likely caused by the setup within the incubation chamber. Because the developmental rate of fish, including *Danio rerio*, is known to be highly influenced by temperature, these differences in temperature would explain why the percentage of hatched embryos in plates handled by the robot was slightly lower [25].

Based on these results we assume that the pipetting robot is suitable to perform fish embryo toxicity (FET) tests in an automated manner, while ensuring validity of the experiments according to the test guidelines.

### Fish embryo toxicity (FET) test

Permethrin and cadmium chloride were investigated in the fish embryo toxicity (FET) test. In this context, it was of particular interest whether the concentration-response relationships for the tested chemicals would differ between (a) the commonly used static exposure scenario, (b) the manually performed semi-static exposure with daily full medium exchanges and (c) the semi-static exposures with automated 50% exchange of media every 1h by use of the pipetting robot.

#### Permethrin

No elevated mortality within the tested concentration range was observed during 48h in the experiments with permethrin (data not shown). This is consistent with data from other studies using embryos of zebrafish and Japanese medaka, respectively [26, 27]. On the contrary, permethrin shows a strong acute toxicity in adult fish [28]; the lower acute embryotoxicity has been attributed to a low availability of permethrin to embryos compared to adult fish [27]. The LC_50_ for permethrin for adult zebrafish after 96h exposure to permethrin is reported as 2.5 μg L^-1^ [29], and 16 μg L^-1^ for fathead minnow [30], and 11 μg L^-1^ for 30 d [31]. It has been hypothesized that these great differences in acute toxicity of permethrin for embryos and adult fish might result from differences in respirational physiology [27]. In adult fish, reduced locomotion due to the neurotoxic effects of permethrin reduces the ventilation rate and consequently the oxygen supply *via* the gills, which may lead to death [32]. In contrast, fish embryos mainly respire oxygen through diffusion [33] and therefore, reduced locomotion due to the neurotoxic effects of permethrin may not be lethal.

Due to the absence of acute toxicity, embryos were exposed for a prolonged exposure time up to 120 hpf and the occurrence of convulsions, ranging from convulsions of the pectoral fins to whole-body spasms, and impaired swimming, meaning that the embryos were no longer able to maintain a normal, upright body posture, were investigated instead. The severity of this effect ranged from occasional loss of the upright body posture and lateral tumbling to the complete loss of upright body posture. Most larvae showing impaired swimming behavior were also affected by convulsions (data not shown).

**Fig 5:**
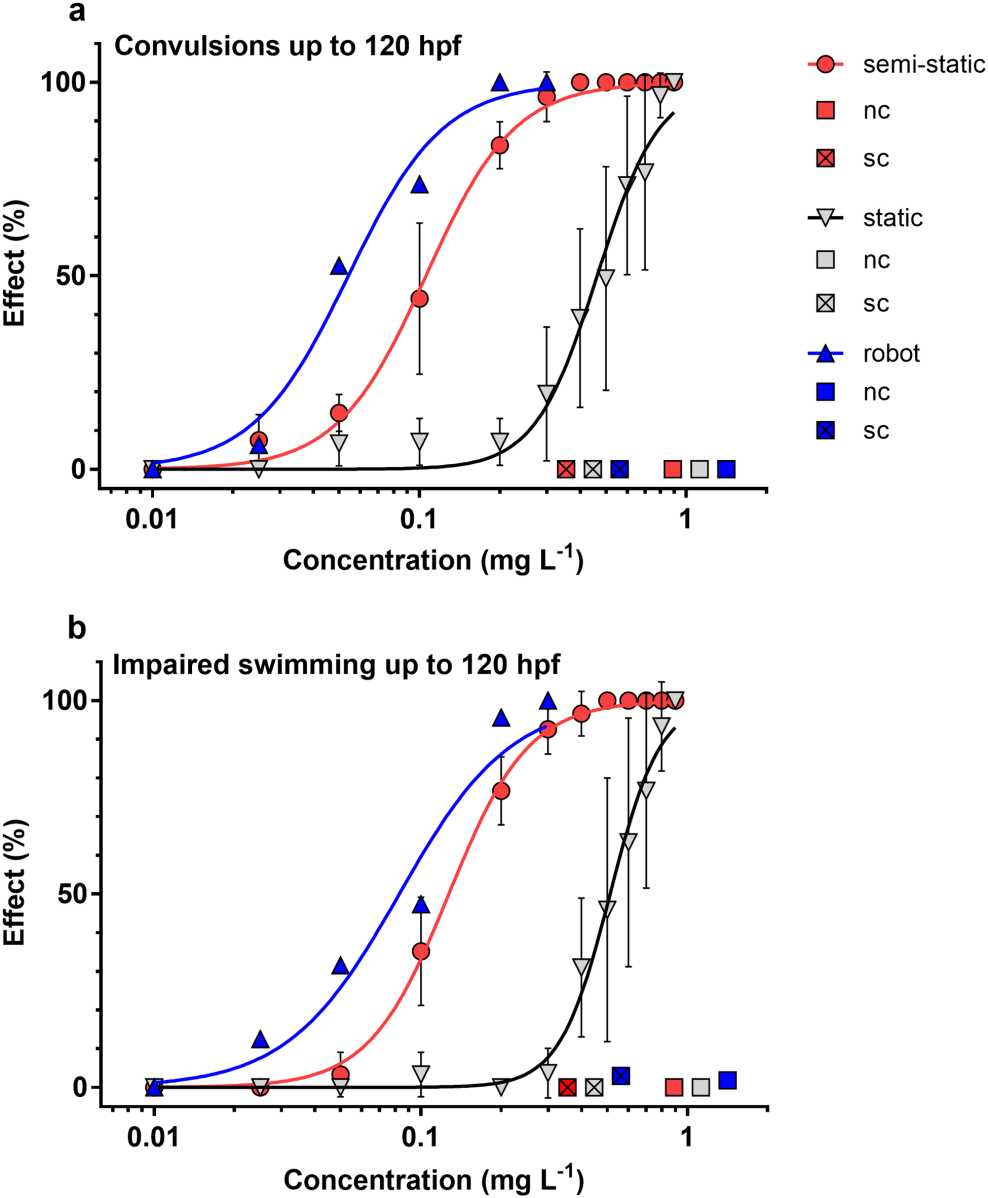
Concentration-dependent effects (in % of exposed zebrafish embryos) of exposure to permethrin up to 120 hpf. Observed sub-lethal effects were convulsions and impaired swimming. Concentration-response curves are provided for static exposure (grey), manual semi-static exposure (red) and automated semi-static exposure using the pipetting robot (blue). nc: negative control; sc: solvent control (0.1% DMSO). Dots indicate mean ± standard deviation from *n* = 3 independent replicates for the static and semi-static exposure, while the concentration-response curves for groups exposed in the robot were assembled from separate tests for each concentration.

The EC_50_ for the occurrence of convulsions was 0.46 mg L^-1^ using static exposure, 0.11 mg L^-1^ for manual semi-static exposure, and lowest for exposure using the pipetting robot at 0.05 mg L^-1^, corresponding to a 4.2- and 9.2-fold difference compared to static exposure (Fig 5). The slopes of all three concentration-response curves were almost equal, and no significant effects were observed in negative and solvent controls. This indicates that differences in observed effects may be mostly attributed to differences in the available concentration of permethrin, ultimately leading to differences in internal concentrations in the embryos. The observed frequency of convulsions was significantly greater in embryos exposed using the pipetting robot compared to manually performed semi-static exposure at three concentrations (0.05, 0.1 and 0.2 mg L^-1^) that were tested for statistical differences (χ2-test, *p* ≤ 0.05).

The concentration-response curves for impaired swimming behavior were similar to those of convulsions, but this endpoint was slightly less sensitive (Fig 5). The EC_50_ was 0.51 mg L^-1^ for static exposure, 0.13 mg L^-1^ for semi-static exposure and 0.08 mg L^-1^ for exposure using the pipetting robot, corresponding to a 3.9- and 6.4-fold difference compared to static exposure. The slopes of the static- and semi-static concentration-response curves were approximately equal, while that of the robot was less steep. The occurrence of impaired swimming behavior was significantly greater for larvae exposed using the pipetting robot compared to manually performed semi-static exposure only at 0.05 mg L^-1^ (χ2-test, *p* ≤ 0.05).

Within some variation, both endpoints were also reported to be caused by permethrin in other studies: a dose-dependent increase in spasms has been observed for 96 h zebrafish embryos (LOEC 50 μg L^-1^) [34] and a concentration of 100 μg L^-1^ resulted in trembling of zebrafish larvae after 120 h [27]. Permethrin also caused trembling movements in medaka larvae [26], the loss of equilibrium in 30 day old medaka [31], loss of equilibrium in adult zebrafish [29] and impaired swimming ability in larvae of fathead minnow [35].

#### Cadmium chloride

**Fig 6:**
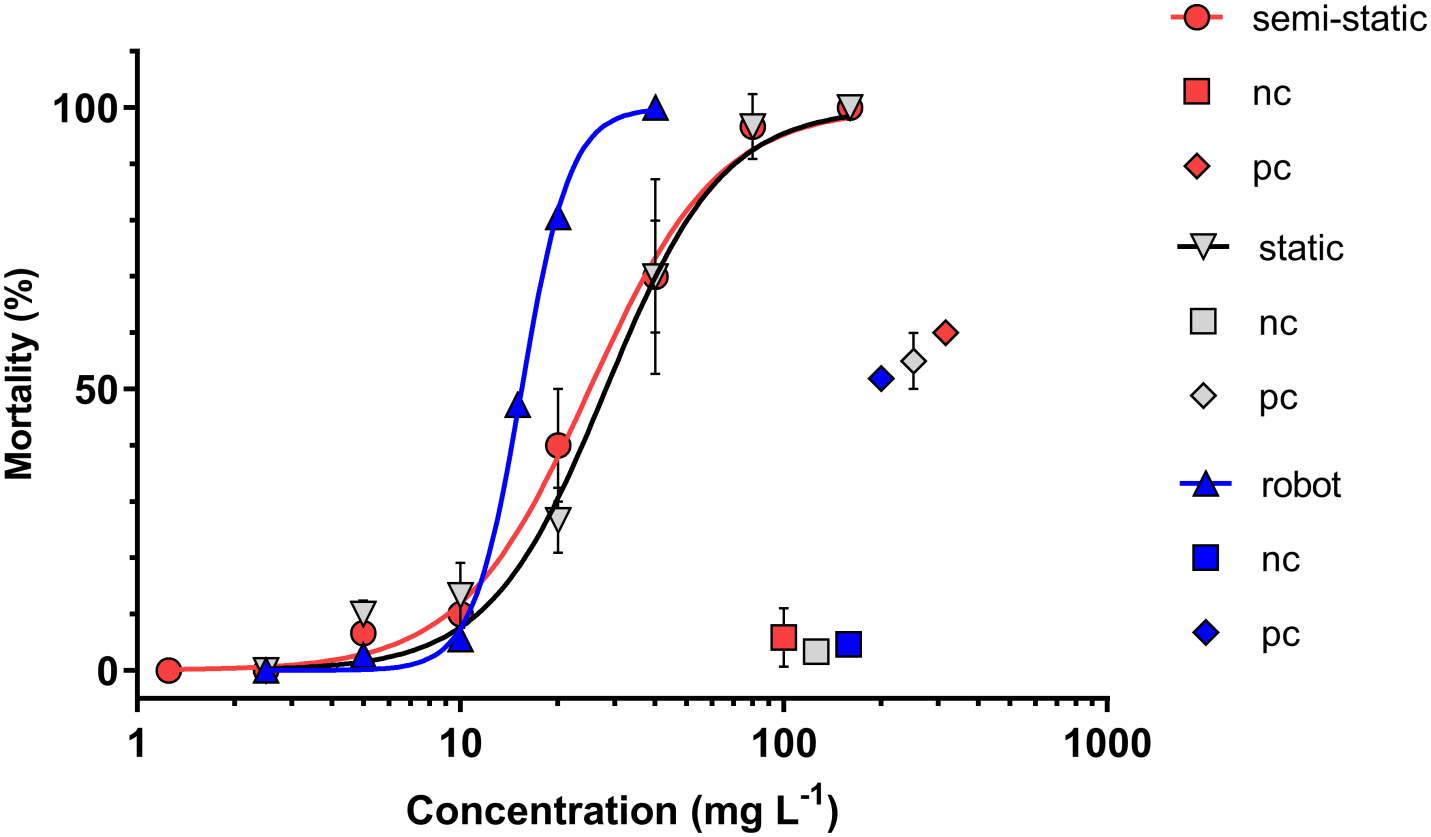
Concentration-dependent mortality (% of exposed zebrafish embryos) after 48 h exposure to cadmium chloride. Concentration-response curves are provided for static exposure (grey), manual semi-static exposure (red) and automated semi-static exposure using the pipetting robot (blue). nc: negative control; pc: positive control (DCA). Dots indicate mean ± standard deviation from *n* = 3 independent replicates for the static and semi-static exposure, while the concentration-response curves for groups exposed using the robot were assembled from separate tests for each concentration.

Mortality was observed in zebrafish embryos exposed to cadmium chloride after 48 h exposure. Notably, no sub-lethal effects were observed in the embryos of any treatment, and the only observed mortality criterion was coagulation (Fig 6). For the static and manual semi-static exposure designs, shape and slope of the concentration-response curves were very similar, with overlapping standard deviations. The resulting LC_50_ for static exposure was 17.2 mg Cd L^-1^ and the LC_50_ for manual semi-static exposure was 15.4 mg Cd L^-1^. A significantly lower LC_50_ resulted from exposure using the pipetting robot, while the slope of the concentration-response curve was much steeper, indicating that that differences in observed mortality were potentially not only due to differences in availability or uptake. The LC_50_ resulting from exposure using the pipetting robot was 9.46 mg Cd L^-1^. While mortality in all negative controls was below 10%, mortality in the positive controls always exceeded 50%, hence rendering all tests valid according to OECD guideline 236 (2013). Out of the three cadmium chloride concentrations tested for statistical differences in mortality between the manual semi-static exposure and that using the pipetting robot (10, 20 and 40 mg L^-1^), significant differences were observed at 20 and 40 mg L^-1^ (χ2-test, *p* ≤ 0.05).

The LC_50_ values for the static- and semi-static exposure were comparable to previously published values determined using zebrafish embryos, which were 30.1 mg L^-1^ [36] and 14.1 mg L^-1^ [37], respectively.

#### Differences between different exposure strategies

Under static exposure conditions, among other, three important factors may limit the concentration of test chemical that is continuously available for uptake into zebrafish embryos: (a) sorption to and precipitation in multiwell plates, (b) uptake into and retention at the protective membranes that enclose the embryos, and (c) biotransformation in the embryo.

**Fig 7:**
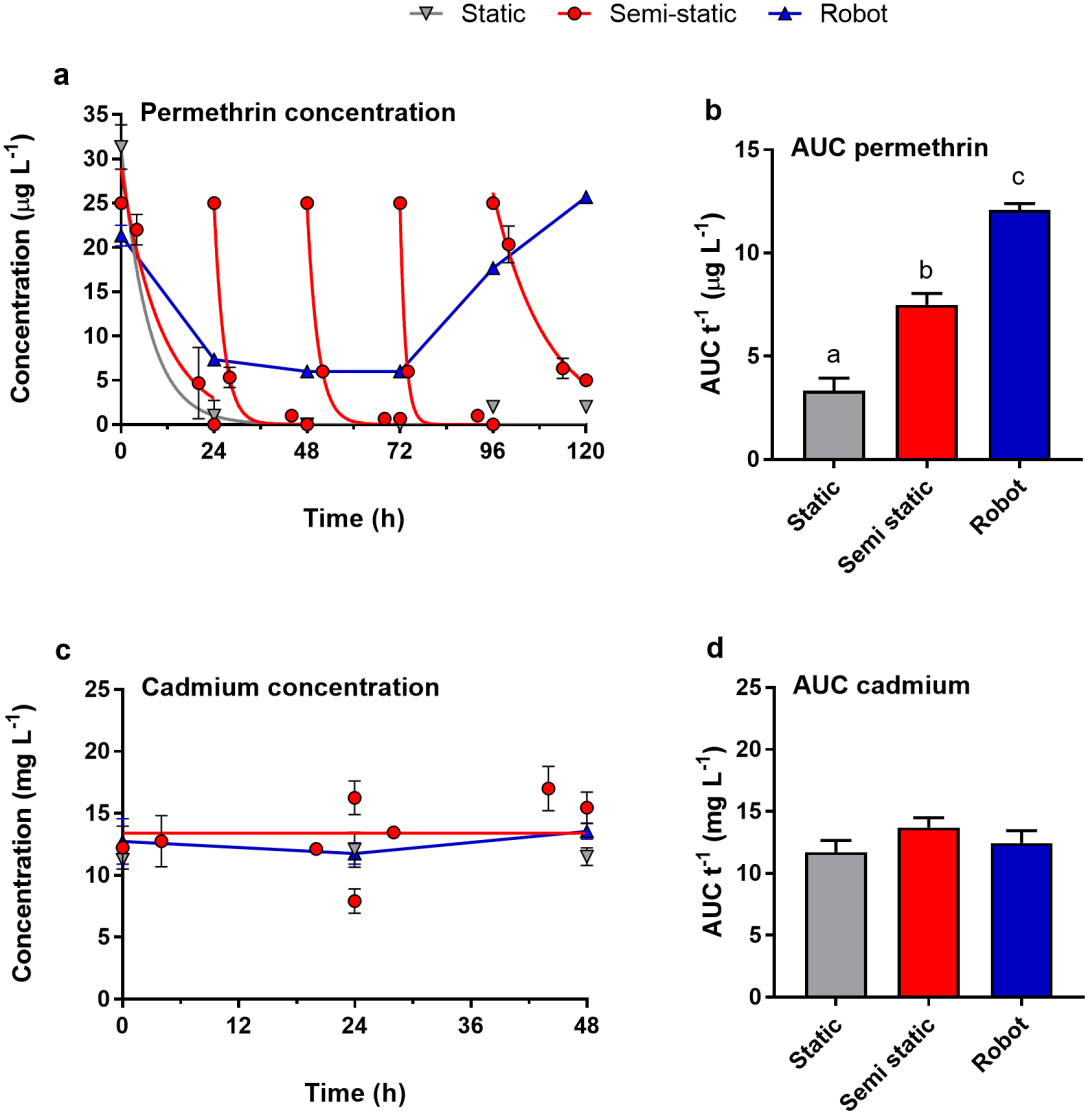
Analytically determined exposure concentrations of permethrin (a) and Cd (c), as well as time-weighted average concentrations (b and d, respectively). Dots indicate mean ± standard deviation from *n* = 3 technical replicates for the three exposure scenarios. Time-weighted average concentrations were calculated by dividing the area under the curve (AUC) by the total exposure time.

Both permethrin and cadmium chloride were tested at comparably low concentrations, and DMSO was used as a solvent carrier for exposure to permethrin. Evaporation and precipitation can thus be assumed to be negligible. Permethrin, on the other hand, is highly lipophilic (*log* K_OW_ 6.5), and the microplates used for exposure consist of polystyrene, which has hydrophobic properties [38]. Binding of permethrin (and to a much lesser extent for cadmium chloride) to the multiwell plates can thus be considered highly relevant. To account for this problem, 24-well plates were saturated with the according exposure concentrations 24 h prior to the test. Given the great differences between the static and semi-static exposure conditions for permethrin, however, and the rapid decrease in analytically determined exposure concentrations (Fig 7) it is obvious that this frequently employed saturation step was still not sufficient.

In addition to losses of permethrin through adsorption to plastic materials, uptake into eggs/ embryos and potentially also biotransformation within the embryo are important processes that lead to a decrease of exposure concentration within the wells of microplates. Thus, more frequent exchanges of exposure solutions should result in more reliable results. Unhatched fish embryos are surrounded by two outer protein membranes: the chorion and the perivitelline membrane [39, 40]. The chorion is a three-layered structure of 10 μm thickness that has numerous pores of 0.17 μm diameter, which have been hypothesized to be responsible for size-dependent restrictions of the uptake of substances [41, 42]. Studies with insecticides have demonstrated the potential of the chorion to modulate toxicity by acting as a physical/ chemical barrier [26]. There is also evidence that this function as a barrier of the chorion increases with lipophilicity of the substance [22]. The chorion also modulates the toxicities of metals: since it is rich in negatively charged glutamic acid, metal cations may selectively bind to such anionic sites [43]. During embryonic development, most metal has been shown to be accumulated in the chorion, a lesser amount in the perivitelline fluid and very little was available for uptake into the embryo itself [44, 45].

Sorption to multiwell plates, uptake into/ retention by the chorion, as well as biotransformation within the embryo potentially deplete the amount of substance in the exposure medium and decrease the concentration available for uptake into the embryo. Any measure to keep exposure concentrations as stable as possible will thus maximize the bioavailable concentration and lead to increased toxicity [46]. The gradual increase in toxicity from static exposure, over manual renewal in 24 h intervals, to automated renewal in 1 h intervals was hypothesized to be a result of an increase in bioavailable concentration. The shorter renewal intervals achieved using the pipetting robot can thus be assumed to getting closer to flow-through exposures, which are generally considered the ‘gold standard’ for exposure to highly lipophilic, volatile or bioaccumulative substances [46].

This hypothesis was verified by chemical analysis of the actual exposure concentrations in the multiwell plates (Fig 7). As expected, concentrations of permethrin rapidly decreased after initiation of static exposures and after each renewal in the semi-static exposure experiments (Fig 7a). No such drastic differences were observed for exposures conducted using the pipetting robot (Fig 7a). These qualitative observations were further substantiated by time-weighted average (TWA) concentrations, which were calculated by dividing the area under the curve by the total exposure time (Fig 7b). TWAs differed significantly between each of the employed exposure methods (one-way ANOVA with Tukey’s post-hoc test, *p* ≤ 0.05), and were least for static, intermediate for semi-static, and greatest for exposures using the robot. No such significant differences were observed for Cd concentrations (Fig 7c and 7d).

#### Potential improvements of the pipetting robot and future directions

Already in its present form, the pipetting robot designed within this study could be a useful addition to the monitoring of drinking and surface water quality. The addition of an automated imaging and scoring system would further decrease the hands-on time and could render the system suitable for autonomous use until embryos have to be exchanged [47]. Depending on the research question, transgenic zebrafish strains, containing, e.g., reporter gene constructs [48], could be used for easier read-out, such as fluorescence measurements. Furthermore, algae, cell lines or microorganisms could be used as biological detection agents instead of zebrafish embryos.

It should be noted that the pipetting robot in its presented configuration was somewhat slower in handling a multiwell plate compared to an experienced laboratory technician; the greatest advantage of the system, however, should be seen in the fact that it can operate continuously with minimal human interaction.

Currently, the pipetting robot is limited to semi-static exchanges of exposure media. By-passing the pipette’s electronics and directly controlling the stepper-motor within the pipette using the Arduino microcontroller board would enable us to vary the pipetting volume between the different steps. This modification would enable testing different concentrations of the investigated sample. This way, more quantitative data could be generated that would allow for well-informed decisions, e.g., when testing sewage treatment effluents. This much more complicated construction, however, was well beyond feasible within the present study.

## Conclusions

In this study, we present a simple low-cost pipetting robot that can be used for performing automated exchanges of exposure media, which renders it suitable for application in online monitoring of drinking or surface water monitoring. The robot was tested through preparing 2-fold serial dilution series, and by conducting experiments with zebrafish embryos exposed to permethrin and cadmium. The accuracy of the pipetting steps was generally high. The apparent toxicity was not only greater in zebrafish embryos exposed to permethrin and cadmium using manual semi-static renewal (24 h interval) compared to static exposure, but also greater in embryos exposed using automated semi-static exposure using the pipetting robot (1 h interval). Thus, we were able to confirm that any attempt to keep exposure concentrations as constant as possible will yield more realistic assessments of toxicity. In this respect, exposure using our pipetting robot can be hypothesized to be similar to flow-through exposure, which is, however, typically more labor- and cost-intensive.

With minor modifications, the presented system can be used in a variety of different setups and environments. Because its construction and operation are very cost-effective and do not require any specialized personnel, provisioning of instructions to replicate this design has makes automation technology accessible to a much higher number of laboratories around the world and has the potential to be a major contribution to the online monitoring of water quality.

## Supporting information

S1 File Supplementary figures. (PDF)

S2 File Arduino sketches. (ZIP)

## References

[1] Rockström J, Steffen W, Noone K, Persson A, Chapin FS, Lambin EF, et al A safe operating space for humanity. Nature. 2009;461(7263):472–5. 10.1038/461472a 19779433

[2] Schwarzenbach RP, Egli T, Hofstetter TB, Gunten Uv, Wehrli B. Global Water Pollution and Human Health. Annual Review of Environment and Resources. 2010;35(1):109–36. 10.1146/annurev-environ-100809-125342

[3] Ostfeld A, Salomons E. Optimal layout of early warning detection stations for water distribution systems security. Journal of Water Resources Planning and Management. 2004;130(5):377–85.

[4] Storey MV, van der Gaag B, Burns BP. Advances in on-line drinking water quality monitoring and early warning systems. Water Research. 2011;45(2):741–7. 10.1016/j.watres.2010.08.049. 20851446

[5] Kramer KJ, Botterweg J. Aquatic biological early warning systems: an overview. Bioindicators and environmental management. 1991:95–126.

[6] Mikol YB, Richardson WR, Van der Schalie WH, Shedd TR, Widder MW. An online real-time biomonitor for contaminant surveillance in water supplies. Journal (American Water Works Association). 2007;99(2):107–15.

[7] Nüßer LK, Skulovich O, Hartmann S, Seiler T-B, Cofalla C, Schuettrumpf H, et al A sensitive biomarker for the detection of aquatic contamination based on behavioral assays using zebrafish larvae. Ecotox Environ Safe. 2016;133:271–80. 10.1016/j.ecoenv.2016.07.033. 27479771

[8] van der Schalie WH, Shedd TR, Knechtges PL, Widder MW. Using higher organisms in biological early warning systems for real-time toxicity detection. Biosensors and Bioelectronics. 2001;16(7–8):457–65. 10.1016/S0956-5663(01)00160-9. 11544040

[9] Bae M-J, Park Y-S. Biological early warning system based on the responses of aquatic organisms to disturbances: A review. Science of The Total Environment. 2014;466–467:635–49. 10.1016/j.scitotenv.2013.07.075. 23962435

[10] Weyers A, Sokull-Klüttgen B, Baraibar-Fentanes J, Vollmer G. Acute toxicity data: a comprehensive comparison of results of fish, Daphnia, and algae tests with new substances notified in the European Union. Environmental toxicology and chemistry. 2000;19(7):1931–3.

[11] Altenburger R, Ait-Aissa S, Antczak P, Backhaus T, Barceló D, Seiler T-B, et al Future water quality monitoring—Adapting tools to deal with mixtures of pollutants in water resource management. Science of The Total Environment. 2015;512–513:540–51. 10.1016/j.scitotenv.2014.12.057. 25644849

[12] Wernersson A-S, Carere M, Maggi C, Tusil P, Soldan P, James A, et al The European technical report on aquatic effect-based monitoring tools under the water framework directive. Environmental Sciences Europe. 2015;27(1):7 10.1186/s12302-015-0039-4

[13] Brack W, Dulio V, Ågerstrand M, Allan I, Altenburger R, Brinkmann M, et al Towards the review of the European Union Water Framework management of chemical contamination in European surface water resources. Science of The Total Environment. 2017;576:720–37. 10.1016/j.scitotenv.2016.10.104. 27810758 PMC8281610

[14] Lammer E, Carr GJ, Wendler K, Rawlings JM, Belanger SE, Braunbeck T. Is the fish embryo toxicity test (FET) with the zebrafish (Danio rerio) a potential alternative for the fish acute toxicity test? Comparative Biochemistry and Physiology Part C: Toxicology & Pharmacology. 2009;149(2):196–209. 10.1016/j.cbpc.2008.11.006. 19095081

[15] Strähle U, Scholz S, Geisler R, Greiner P, Hollert H, Rastegar S, et al Zebrafish embryos as an alternative to animal experiments—A commentary on the definition of the onset of protected life stages in animal welfare regulations. Reprod Toxicol. 2012;33(2):128–32. 10.1016/j.reprotox.2011.06.121. 21726626

[16] DIN 38415–6:2003–08. German standard methods for the examination of water, waste water and sludge—subanimal testing (group T). Part 6. Toxicity to fish; determination of the non-acute-poisonous effect of waste water to fish eggs by dilution limits (T 6).

[17] OECD. Test No. 236: Fish Embryo Acute Toxicity (FET) Test: OECD Publishing.

[18] Vincze K, Graf K, Scheil V, Köhler H-R, Triebskorn R. Embryotoxic and proteotoxic effects of water and sediment from the Neckar River (Southern Germany) to zebrafish (Danio rerio) embryos. Environmental Sciences Europe. 2014;26(1):3 10.1186/2190-4715-26-3

[19] Pearce JM. Materials science. Building research equipment with free, open-source hardware. Science. 2012;337(6100):1303–4. Epub 2012/09/18. 10.1126/science.1228183 . 22984059

[20] Nagel R. DarT: The embryo test with the zebrafish Danio rerio—A general model in ecotoxicology and toxicology. ALTEX. 2002;19:38–48. 12096329

[21] Hollert H, Keiter S, Koenig N, Rudolf M, Ulrich M, Braunbeck T. A new sediment contact assay to assess particle-bound pollutants using zebrafish (Danio rerio) embryos. Journal of Soils and Sediments. 2003;3(3):197–207. 10.1065/jss2003.09.085

[22] Braunbeck T, Boettcher M, Hollert H, Kosmehl T, Lammer E, Leist E, et al Towards an alternative for the acute fish LC_50_ test in chemical assessment: The zebrafish (*Danio rerio*) embryo toxicity test—an update. ALTEX. 2005;22(2):87–102. 15953964

[23] Lammer E, Carr GJ, Wendler K, Rawlings JM, Belanger SE, Braunbeck T. Is the fish embryo toxicity test (FET) with the zebrafish (*Danio rerio*) a potential alternative for the fish acute toxicity test? Comparative Biochemistry and Physiology Part C: Toxicology & Pharmacology. 2009;149(2):196–209. 19095081 10.1016/j.cbpc.2008.11.006

[24] European Commission. The European Parliament and the European Council Directive 2010/63/EU of the 22 September 2010 on the protection of animals used for scientific purposes. Official Journal of the European Union. 2010: L276/33.

[25] Kimmel CB, Ballard WW, Kimmel SR, Ullmann B, Schilling TF. Stages of embryonic development of the zebrafish. Developmental Dynamics. 1995;203(3):253–310. 10.1002/aja.1002030302 8589427

[26] Gonzalez-Doncel M, de la Pena E, Barrueco C, Hinton DE. Stage sensitivity of medaka (Oryzias latipes) eggs and embryos to permethrin. Aquatic Toxicology. 2003;62(3):255–68. 10.1016/s0166-445x(02)00090-5 12560173

[27] Knobel M, Busser FJM, Rico-Rico A, Kramer NI, Hermens JLM, Hafner C, et al Predicting Adult Fish Acute Lethality with the Zebrafish Embryo: Relevance of Test Duration, Endpoints, Compound Properties, and Exposure Concentration Analysis. Environmental Science & Technology. 2012;46(17):9690–700. 10.1021/es301729q 22835061

[28] Hill IR. Aquatic organisms and pyrethroids. Pesticide Science. 1989;27(4):429–57. 10.1002/ps.2780270408

[29] Zhang Z-Y, Yu X-Y, Wang D-L, Yan H-J, Liu X-J. Acute toxicity to zebrafish of two organophosphates and four pyrethroids and their binary mixtures. Pest Management Science. 2010;66(1):84–9. 10.1002/ps.1834 19728319

[30] Russom CL, Bradbury SP, Broderius SJ, Hammermeister DE, Drummond RA. Predicting modes of toxic action from chemical structure: Acute toxicity in the fathead minnow (Pimephales promelas). Environmental Toxicology and Chemistry. 1997;16(5):948–67. 10.1002/etc.2249 23733666

[31] Rice PJ, Drewes CD, Klubertanz TM, Bradbury SP, Coats JR. Acute toxicity and behavioral effects of chlorpyrifos, permethrin, phenol, strychnine, and 2,4-dinitrophenol to 30-day-old Japanese medaka (Oryzias latipes). Environmental Toxicology and Chemistry. 1997;16(4):696–704.

[32] Haya K. TOXICITY OF PYRETHROID INSECTICIDES TO FISH. Environmental Toxicology and Chemistry. 1989;8(5):381–91. 10.1897/1552-8618(1989)8[381:topitf]2.0.co;2

[33] Rombough P. Gills are needed for ionoregulation before they are needed for O-2 uptake in developing zebrafish, Danio rerio. Journal of Experimental Biology. 2002;205(12):1787–94. 12042337 10.1242/jeb.205.12.1787

[34] DeMicco A, Cooper KR, Richardson JR, White LA. Developmental Neurotoxicity of Pyrethroid Insecticides in Zebrafish Embryos. Toxicological Sciences. 2010;113(1):177–86. 10.1093/toxsci/kfp258 19861644 PMC2794336

[35] Floyd EY, Geist JP, Werner I. Acute, sublethal exposure to a pyrethroid insecticide alters behavior, growth, and predation risk in larvae of the fathead minnow (Pimephales promelas). Environmental Toxicology and Chemistry. 2008;27(8):1780–7. 10.1897/07-448.1 18380520

[36] Hallare AV, Schirling M, Luckenbach T, Kohler HR, Triebskorn R. Combined effects of temperature and cadmium on developmental parameters and biomarker responses in zebrafish (Danio rerio) embryos. Journal of Thermal Biology. 2005;30(1):7–17. 10.1016/j.jtherbio.2004.06.002

[37] Redelstein R, Zielke H, Spira D, Feiler U, Erdinger L, Zimmer H, et al Bioaccumulation and molecular effects of sediment-bound metals in zebrafish embryos. Environmental Science and Pollution Research. 2015;22(21):16290–304. 10.1007/s11356-015-5328-3 26354112

[38] Palmgren JJ, Monkkonen J, Korjamo T, Hassinen A, Auriola S. Drug adsorption to plastic containers and retention of drugs in cultured cells under in vitro conditions. European Journal of Pharmaceutics and Biopharmaceutics. 2006;64(3):369–78. 10.1016/j.ejpb.2006.06.005 16905298

[39] Mizell M, Romig ES. The aquatic vertebrate embryo as a sentinel for toxins: zebrafish embryo dechorionation and perivitelline space microinjection. Int J Dev Biol. 1997;41(2):411–23. 9184351

[40] Scholz S, Fischer S, Guendel U, Kuester E, Luckenbach T, Voelker D. The zebrafish embryo model in environmental risk assessment—applications beyond acute toxicity testing. Environmental Science and Pollution Research. 2008;15(5):394–404. 10.1007/s11356-008-0018-z 18575912

[41] Bonsignorio D, Perego L, DelGiacco L, Cotelli F. Structure and macromolecular composition of the zebrafish egg chorion. Zygote. 1996;4(2):101–8. 8913023 10.1017/s0967199400002975

[42] Cheng JP, Flahaut E, Cheng SH. Effect of carbon nanotubes on developing zebrafish (Danio rerio) embryos. Environmental Toxicology and Chemistry. 2007;26(4):708–16. 10.1897/06-272r.1 17447555

[43] Rombough PJ. THE INFLUENCE OF THE ZONA RADIATA ON THE TOXICITIES OF ZINC, LEAD, MERCURY, COPPER AND SILVER IONS TO EMBRYOS OF STEELHEAD TROUT SALMO-GAIRDNERI. Comparative Biochemistry and Physiology C-Pharmacology Toxicology & Endocrinology. 1985;82(1):115–7. 10.1016/0742-8413(85)90216-6 2865049

[44] Burnison BK, Meinelt T, Playle R, Pietrock M, Wienke A, Steinberg CEW. Cadmium accumulation in zebrafish (Danio rerio) eggs is modulated by dissolved organic matter (DOM). Aquatic Toxicology. 2006;79(2):185–91. 10.1016/j.aquatox.2006.06.010 16854477

[45] Jezierska B, Ługowska K, Witeska M. The effects of heavy metals on embryonic development of fish (a review). Fish Physiology and Biochemistry. 2008;35(4):625–40. 10.1007/s10695-008-9284-4 19020985

[46] Traas T, Van Leeuwen C. Ecotoxicological effects. Risk Assessment of Chemicals: Springer; 2007 p. 281–356.

[47] Alshut R, Legradi J, Liebel U, Yang L, van Wezel J, Strähle U, et al Methods for Automated High-Throughput Toxicity Testing Using Zebrafish Embryos In: Dillmann R, Beyerer J, Hanebeck UD, Schultz T, editors. KI 2010: Advances in Artificial Intelligence: 33rd Annual German Conference on AI, Karlsruhe, Germany, September 21–24, 2010 Proceedings. Berlin, Heidelberg: Springer Berlin Heidelberg; 2010 p. 219–26.

[48] Legler J, Zeinstra LM, Schuitemaker F, Lanser PH, Bogerd J, Brouwer A, et al Comparison of in vivo and in vitro reporter gene assays for short-term screening of estrogenic activity. Environmental science & technology. 2002;36(20):4410–5. 12387416 10.1021/es010323a

