# Supplementary material for: A versatile and low-cost open source pipetting robot for automation of toxicological and ecotoxicological bioassays
Source: PLoS One. 2017 Jun 16;12(6):e0179636. doi: 10.1371/journal.pone.0179636 (PMC5473567; doi:10.1371/journal.pone.0179636)
Supplement: S1 File — (PDF) [file pone.0179636.s001.pdf]

SUPPLEMENTARY INFORMATION

A versatile and low-cost open source pipetting robot for automation of toxicological and ecotoxicological bioassays

Sebastian Steffens, Leonie Nüßer, Thomas-Benjamin Seiler, Nadine Ruchter, Mark Schumann, Ricarda Döring, Catrina Cofalla, Avi Ostfeld, Elad Salomons, Holger Schüttrumpf, Henner Hollert, Markus Brinkmann*

Total page number: six, including one table

and four figures.

*Dr. Markus Brinkmann (corresponding author)

44 Campus Drive

Saskatoon SK, S7N 5B3

Phone: +1 (306) 966 1204

markus.brinkmann@usask.ca

Table A: List of components for construction of the Arduino-controlled pipetting robot used throughout the present study.

Open Beam USA	
L-bracket	28 pcs.
T-bracket	19 pcs.
NEMA mount adapter	1 pc.
Bracket NEMA 17 stepper motor	2 pcs.
Aluminum extrusion profile (30 mm)	4 pcs.
Aluminum extrusion profile (60 mm)	8 pcs. with custom M3 thread to attach baseplate to unit; 1 pc. with two custom M4 threads to attach timing belt to the moving platform
Aluminum extrusion profile (90 mm)	13 pcs.
Aluminum extrusion profile (120 mm)	9 pcs.
Aluminum extrusion profile (150 mm)	7 pcs.
Adafruit.com	
Stepper motor NEMA, 200 steps, 12V, 350 mA	1 pc.
Spindle drive, 24 V	1 pc.
Linear bearing platform, small	4 pcs.
Linear bearing platform, large	4 pcs.
Aluminum timing pulley, 6mm belt, 20 tooth, 5 mm bore	1 pc.
Linear rail shaft support, 8 mm diameter	8 pcs.
Hardened rail shaft, 8 mm diameter, 425mm	2 pcs.
Hardened rail shaft, 8 mm diameter, 135mm	2 pcs.
Timing belt, 2 mm pitch, 6 mm width, 1,164 mm length	1 pc.
Custom-built parts	
Baseplate	1 pc. (Figure S1)
Customized pipette receptacle	1 pc. (Figure S2)
Moving platform	1 pc. (Figure S3)
Sample intake and outflow	1 pc. (Figure S4)

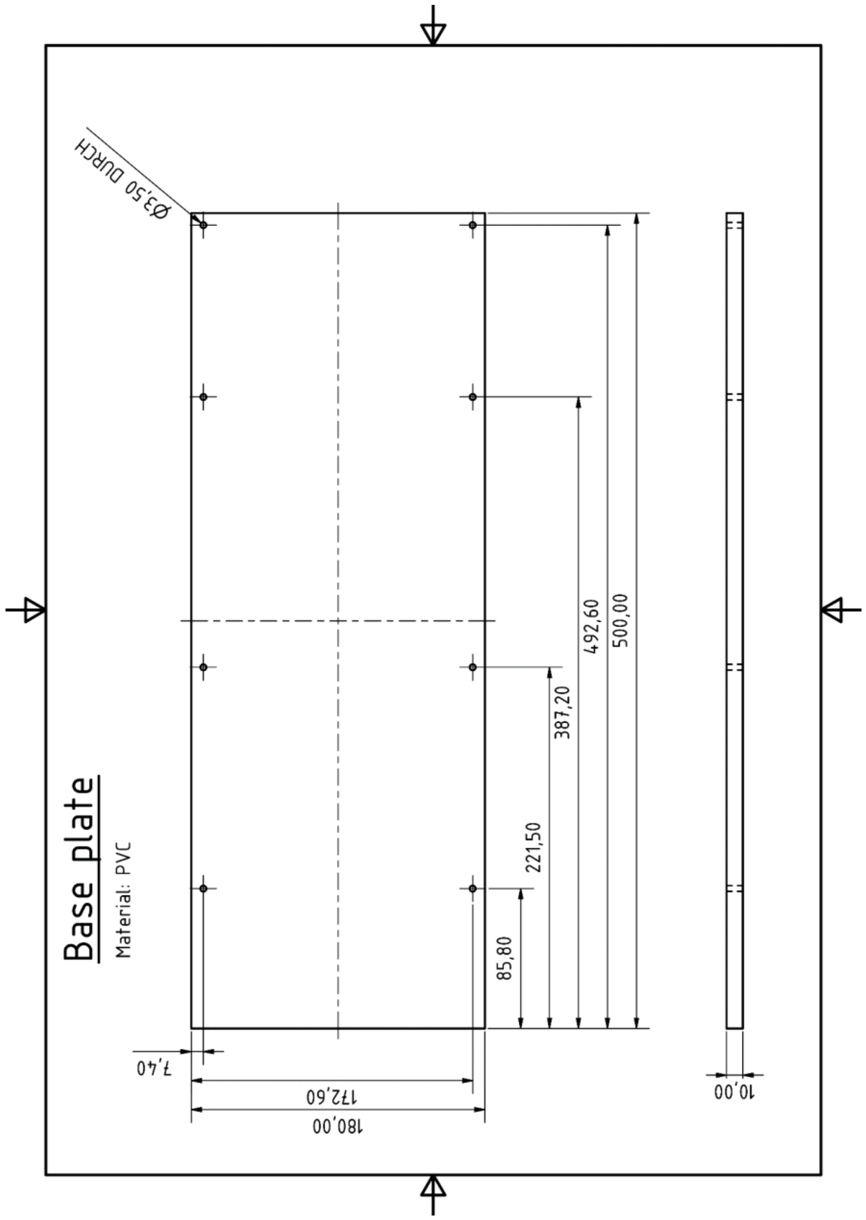

Fig A: Technical drawing of the baseplate. The baseplate of the pipetting robot was custom-made from 10 mm PVC sheet material. All dimensions are provided in mm.

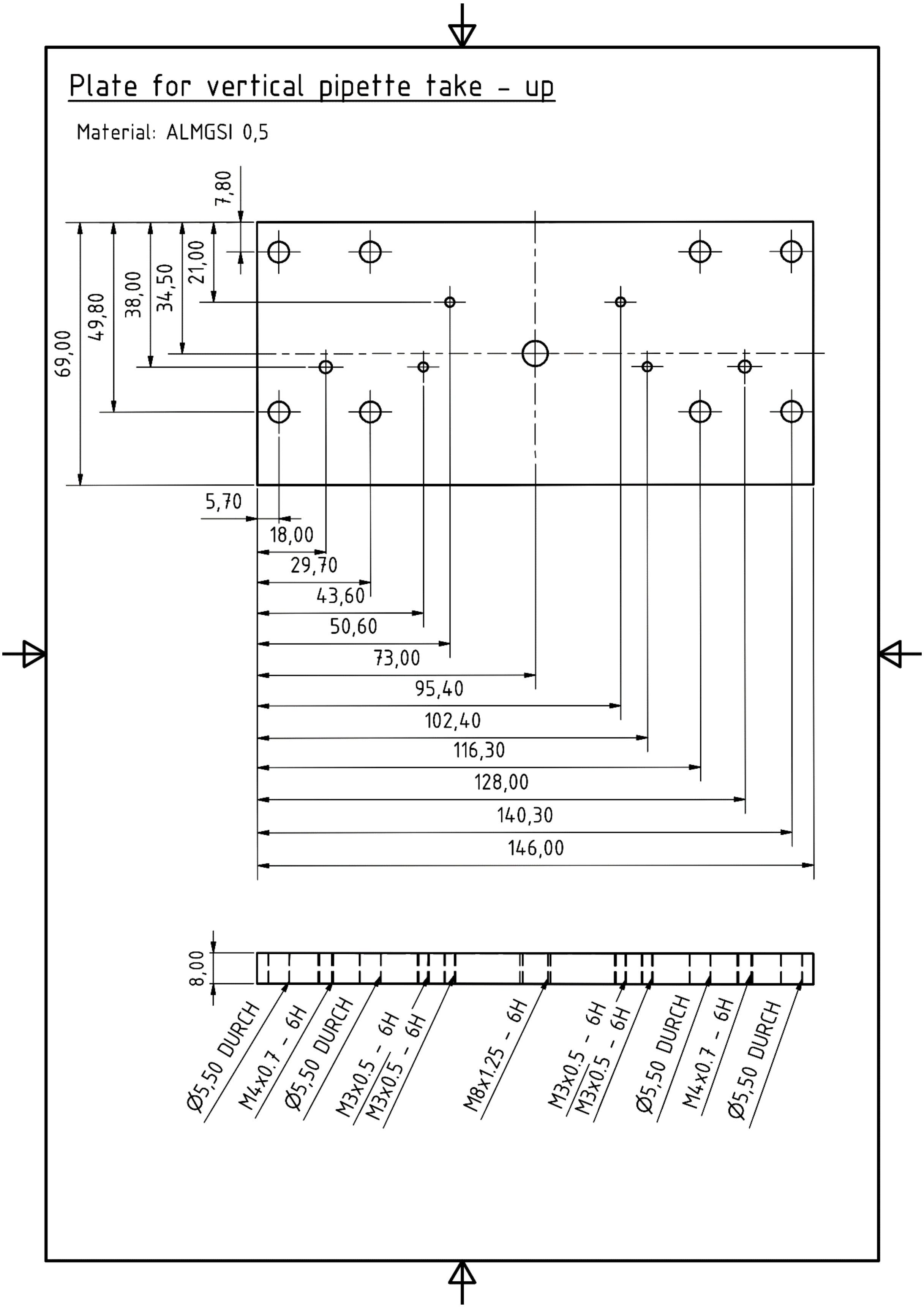

Fig B: Technical drawing of the pipette receptacle. The receptacle was custom-made from 8 mm aluminum sheet material. All dimensions are provided in mm.

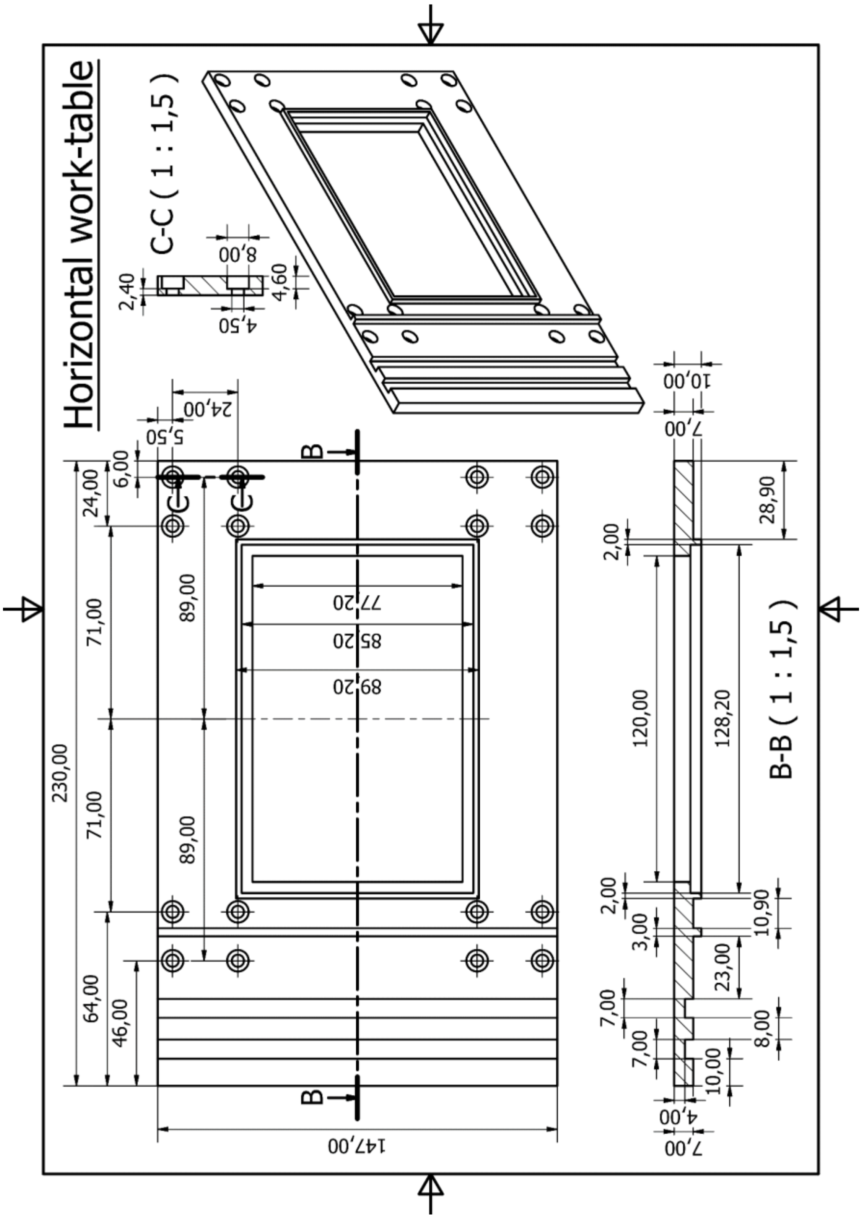

Fig C: Technical drawing of the moving platform. The moving platform was custom-made from 11 mm aluminum sheet material. All dimensions are provided in mm.

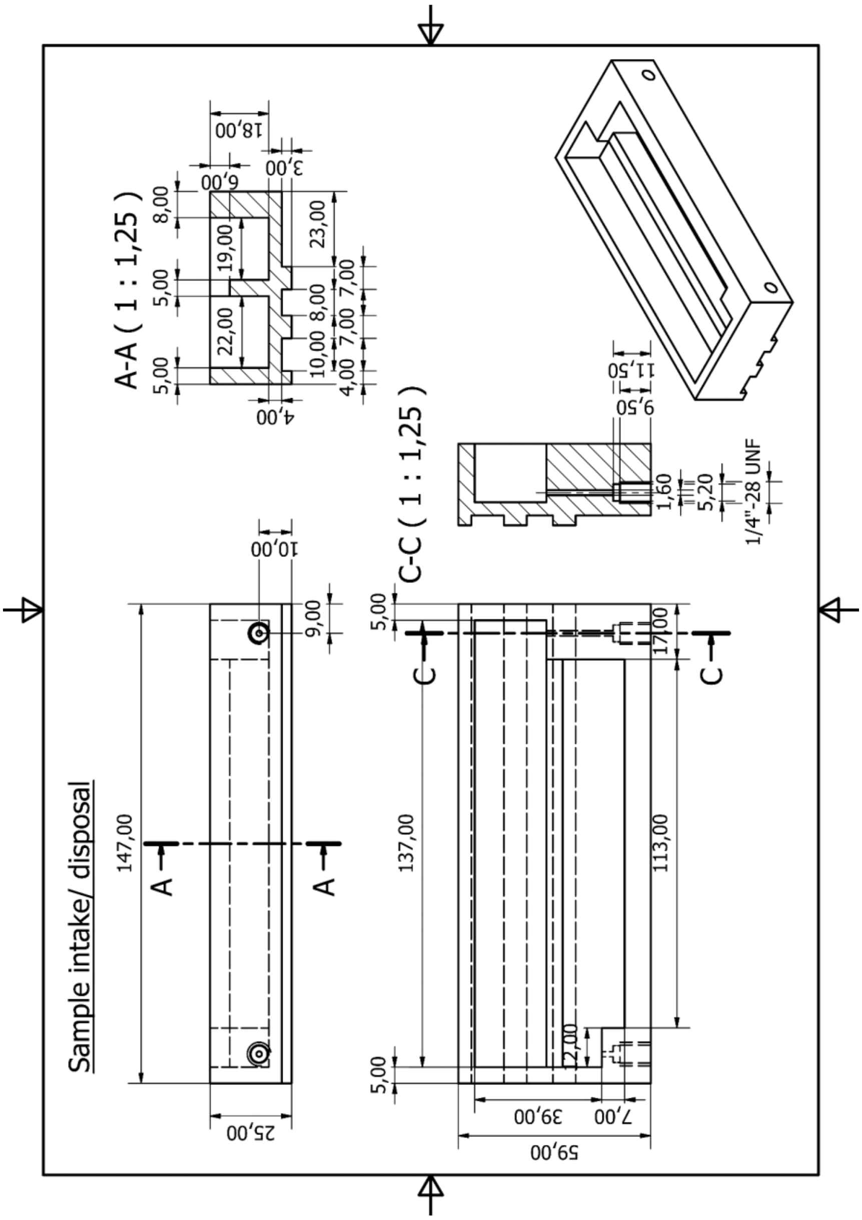

Fig D: Technical drawing of the sample intake and outflow. The sample intake and outflow was custom-made from bulk PTFE material. All dimensions are provided in mm.
