# Supplementary figures and images for: A versatile and low-cost open source pipetting robot for automation of toxicological and ecotoxicological bioassays
Source: PLoS One. 2017 Jun 16;12(6):e0179636. doi: 10.1371/journal.pone.0179636 (PMC5473567; doi:10.1371/journal.pone.0179636)

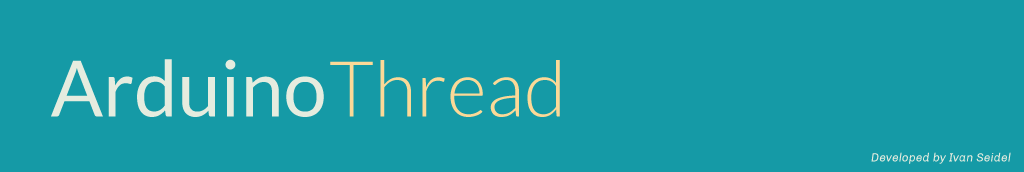

Supplement: S2 File — (ZIP) [file pone.0179636.s002.zip › Steffens and Nuesser et al. Arduino sketches/sketch_oct04c/libraries/ArduinoThread-master/images/ArduinoThread.png]
